# Laser Photobiomodulation for a Complex Patient with Severe Hydroxyurea-Induced Oral Ulcerations

**DOI:** 10.1155/2016/9810480

**Published:** 2016-11-10

**Authors:** Marco Cabras, Adriana Cafaro, Alessio Gambino, Roberto Broccoletti, Ercole Romagnoli, Davide Marina, Paolo G. Arduino

**Affiliations:** ^1^Department of Surgical Sciences, CIR-Dental School, University of Turin, Turin, Italy; ^2^Department of Surgical and Diagnostic Sciences (DISC), University of Genoa, Genoa, Italy; ^3^Private Practice, Milan, Italy

## Abstract

Patients affected by polycythemia vera (PV), a myeloproliferative neoplasm characterized by an elevated red blood cell mass, are at high risk of vascular and thrombotic complications. Conventional therapeutic options aim at reducing vascular and thrombotic risk; low-dose aspirin and phlebotomy are first-line recommendations, for patients at low risk of thrombotic events, whereas cytoreductive therapy, usually hydroxyurea (HU) or interferon alpha, is recommended for high-risk patients. In the present study, we report the case of a patient with persistent oral ulcerations, possibly related to long-lasting HU treatment, firstly treated with topic and systemic corticosteroids and then more effectively with the addition of low-level laser therapy. Laser photobiomodulation has achieved pain control and has contributed to the healing of oral ulcers without any adverse effect; this has permitted a reduction in the dose of systemic corticosteroids and the suspension of the use of the topic ones, due to the long-term stability of oral health, even after the interruption of low-level laser therapy sessions.

## 1. Introduction

Patients affected by polycythemia vera (PV), a myeloproliferative neoplasm characterized by an elevated red blood cell mass, are at high risk of vascular and thrombotic complications; they also have reduced quality of life due to a substantial symptom burden that includes pruritus, fatigue, constitutional symptoms, microvascular disturbances, and bleeding. Conventional therapeutic options aim at reducing vascular and thrombotic risk; low-dose aspirin and phlebotomy are first-line recommendations, for patients at low risk of thrombotic events, whereas cytoreductive therapy, usually hydroxyurea (HU) or interferon alpha, is recommended for high-risk patients [1].

Mucocutaneous ulcers are possible complicating adverse effects caused by HU; these lesions can appear right after the beginning of HU therapy or can be a later effect, with similar clinical presentation in both situations. The oral alterations are uncommon, but they could have a greater clinical impact because of severe pain and feeding or speaking impairment [2, 3].

Data from literature suggest that oral ulceration is the first occurring oral side effect of hydroxyurea, usually developing after a variable period of time since administration, ranging between 5 months and up to 3 years. Mucocutaneous lesions have been diagnosed in 167 of 3411 patients on hydroxyurea with Philadelphia-chromosome-negative chronic myeloproliferative neoplasms in a large Italian cohort [4]; of this subgroup, 27 patients presented oral ulcerations. Almost half of these patients needed to discontinue hydroxyurea with resolution of lesions in about one month. The remaining patients received local therapy, consisting of mouthwash with folic acid and vitamin A, and they obtained some symptomatic improvement. However, complete healing was achieved only after HU dose reduction or suspension in an average time frame of three months.

Other studies [5, 6] have reported 12.5% to 13% prevalence of mucocutaneous involvement in smaller cohorts of 40 and 158 patients, respectively, including mucosal symptoms, such as pain and burning sensation, atrophy, and ulcers.

Actually, dose reduction or suspension of HU, if possible, can be considered as the most effective available measure [2, 4–6].

In the present study, we report the case of a patient with persistent oral ulcerations, possibly related to long-lasting HU treatment, firstly treated with topic and systemic corticosteroids and then more effectively with the addition of low-level laser therapy.

## 2. Case Presentation

**Figure 1:**
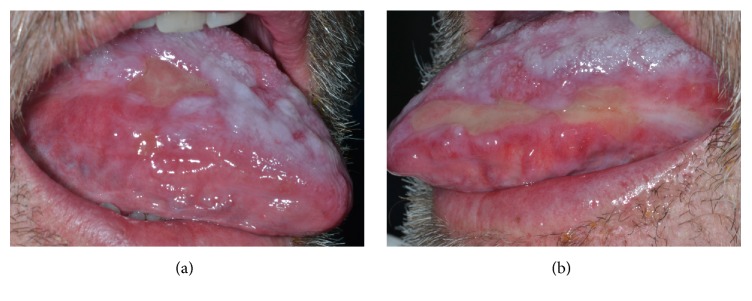
Erosive lesions presented in (a) and (b) lateral border of the tongue.

A 72-year-old male (diagnosed with PV in 1994 and treated with HU since 1995) was referred to the Oral Medicine Section of the Turin University in March 2014 because of severe tongue and lips pain and difficulty in feeding and speaking. At the time being, HU dosage consisted of three 500 mg tablets daily. At physical examination, extensive ulcerations on the tongue and lips were observed (Figure 1). Based on the clinical appearance and the topography of the lesions, a differential diagnosis between HU related ulcers and erosive lichen was posed. However, advice against the execution of the oral biopsy came directly from the oncologist, due to the very high level of platelets (more than 1.000.000/*μ*L) detected in the patient's blood.

Due to the impossibility of solving the diagnostic dilemma through histopathological examination, we opted for an* ex juvantibus* approach and proceeded to administer an appropriate therapy with the intention of improving at first the patient's quality of life.

In agreement with the oncologist, a combined treatment with systemic (oral administration of 75 mg of prednisone daily) and topic corticosteroids (clobetasol 0.05% ointment twice daily) was established. During the following two months, a reduction of symptoms and oral lesions allowed a therapy reduction to 12.5 mg daily of prednisone, with the topic treatment being unchanged.

After a short period of relative wellness, in July 2014 the oral situation worsened again, with diffuse extensive oral ulcerations, high levels of pain, and very poor quality of life, although HU dosage had been unaltered since our first encounter with the patient, four months before. Therapy was modified again: 75 mg of prednisone daily was administered for two weeks and then, in accordance with the oncologist, dosage was reduced to 50 mg daily, but with no reduction of symptoms and pain; tacrolimus ointment (Protopic® 0.1% ointment) was used instead of topical clobetasol, but this was to no avail.

In September 2014, the decision was taken to add photobiomodulation (low-level laser therapy, LLLT) to the pharmacological therapy, with a twice-weekly frequency. A diode laser (GaAlAs) 810 nm (Ora-Laser_D-Light ORALIA Medical GmbH, Germany) was used, with spot size 1.3 cm^2^, power setting 100 mW, mean power 50 mW, duty cycle 50%, frequency 10.000 Hz, fluence 6 J/cm^2^ (calculated as follows: Fluence = power × time/surface), treatment time 160 seconds, point by point technique, not in contact (distance to the mucosa about 2 mm) perpendicular to the treatment area.

Noncontact mode was specifically chosen in order to prevent the onset of additional pain which could be obtained had we preferred the direct contact between the laser probe and the oral ulcers undergoing the LLLT treatment.

After 2 weeks, a marked improvement was immediately observed with disappearance of pain and the beginning of reepithelialization of the mucosa; at that time, HU dosage consisted of three 500 mg tablets daily interchanged with four 500 mg tablets every other day.

However, due to hospital admission for pulmonary complications, LLLT had to be suspended for 2 months, together with a gradual reduction of prednisone dosage until complete suspension: inevitably, the interruption of these treatments caused a worsening of the oral clinical situation.

After another 4 weeks of photobiomodulation treatment sessions, the lesions had not disappeared completely, but a significant reduction of pain was observed. Due to the improvement of the oral condition, the decision was taken to stop topic corticosteroid treatment continuing with systemic treatment and laser therapy, and the remission of the lingual lesions was obtained.

**Figure 2:**
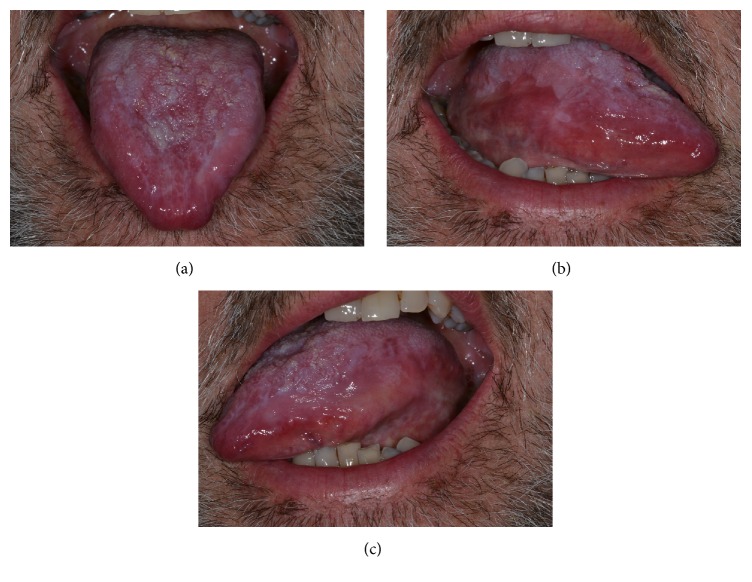
(a–c) Complete disappearance of the erosive tongue lesion at the end of the treatment provided.

After another 3 weeks, symptoms and mucosal lesions disappearance, together with functional recovery, were observed (Figure 2).

Since then, photobiomodulation treatment was concluded, together with the gradual reduction of systemic corticosteroid treatment in the following months. In the meantime, HU dosage had been maintained, at the aforementioned dosage of three to four 500 mg tablets every other day.

In September 2015, HU was finally substituted with JACAVI; since then, the patient continued to be ulcers-free;* ad libitum* application of topical corticosteroid was prescribed to the patient but has never been used. Our last follow-up visit, conducted in July 2016, showed no signs or symptoms at the oral cavity; meanwhile, prednisone was furthermore reduced to a minimum of 5 mg every other day.

## 3. Discussion

PV is a classic Philadelphia-chromosome-negative myeloproliferative neoplasm (MPN), characterized primarily by an increase in red blood cell mass together with an excessive proliferation of myeloid and megakaryocytic components in the bone marrow, resulting in high red blood cells and platelet and white blood cells counts.

In the past 10 years, the pathophysiology of this condition has been defined as JAK/STAT pathway activation, almost always due to mutations in JAK2 exon 12 or 14 (JAK2 V617F) [4]. These findings have become indicative not only for diagnosis, so much that the presence of the JAK2 V617F mutation has been introduced as a major criterion in the diagnosis of PV since 2008, but also for treatment, most recently culminating in the approval of JAK inhibitors as an alternative for patients who are resistant or intolerant to HU [1].

Dresler and Stein described the synthesis of HU in 1869; the antitumor activity of this drug was reported in the 1960s, when it was tested against mouse leukemia and other malignancies. In the 1990s, HU was described as an inhibitor of ribonucleotide reductase, an enzyme involved in the synthesis of deoxyribonucleotides: by depleting the pool of deoxyribonucleotides, HU inhibits DNA synthesis, arresting the cell cycle at the G1/S phase [7].

On the other hand, skin and mucosal side effects are not completely understood and usually occur following long-term therapy; cutaneous abnormalities include xerosis, ichthyosiform lesions, dark brown pigmentation of skin folds and nails, and malleolar ulcers as well as malignant tumors [8–10].

Oral lesions are rare complications that can rapidly develop right after the start of the therapy [2] or as the side effect of a long-term exposure [3]; they often appear as painful ulcers, sometimes associated with skin lesions. Glossitis and stomatitis with intense erythema may also be observed [6, 11].

According to literature, there is no specific protocol for management of HU oral ulcers, apart from the dose reduction or suspension of HU itself. In the largest cohort of patients undergoing HU, a mouthwash containing folic acid and vitamin A was prescribed, obtaining symptomatic improvement; however, no details regarding the methods (dosage, timing, etc.) are reported; moreover, the authors declared that healing was achieved only after HU dose reduction or suspension in an average time frame of three months [4].

To our knowledge, only two cases of oral cancer following long-term treatment with HU have been reported [12, 13].

Therapeutic use of laser light was introduced in 1966, when Endre Mester published the first scientific article concerning stimulating and nonthermal effects of ruby laser on mice skin [14].

The energy of certain wavelengths (635–1100 nm) is absorbed by photoacceptor chromophores in the respiratory chain (cytochrome c oxidase) in mitochondria: this results in a change in the redox status of the cell, together with an increase in ATP production and cell proliferation rate [15–17], thanks to the activation of the nucleic acids, proteins, and enzymes regulation paths. It also participates in the regulation of the level of cytokines and growth factors and inflammation and tissue oxygenation mediators.

These biochemical cell changes lead to advantages as an increase in the speed of wound healing and a marked analgesic effect [15–18].

The rationale of using photobiomodulation is related to the possibility of accelerating the healing process of ongoing oral ulcers, as shown and reported by several studies [19–22].

In this case report laser photobiomodulation has achieved pain control and has contributed to the healing of oral ulcers without any adverse effect; this has permitted a reduction in the dose of systemic corticosteroids and the suspension of the use of the topic ones, due to the long-term stability of oral health, even after the interruption of LLLT sessions.
